# Migration of Tenckhoff Catheter to Sigmoid Colon: A Rare Delayed Complication

**DOI:** 10.1155/2022/5443787

**Published:** 2022-03-11

**Authors:** Uğur Topal, Abdullah Ülkü, Ahmet Gökhan Sarıtaş, Atilgan Tolga Akçam, Ayşe Gizem Ünal, İsmail Cem Eray

**Affiliations:** Department of General Surgery, Cukurova University Faculty of Medicine, 01330 Cukurova/Adana, Turkey

## Abstract

Bowel perforation associated with inserted peritoneal dialysis (PD) catheter mainly occurs during the perioperative period. Delayed bowel perforation is difficult to diagnose because of its different clinical signs and rarity. A 53-year-old woman developed acute abdomen after her PD catheter was changed. It was found that the changed catheter perforated the sigmoid colon. Primary repair of the perforated area of the sigmoid colon was performed, and the last inserted PD catheter was removed. The postoperative period and recovery were uneventful. Perforations due to the PD catheter may remain silent until the catheter is replaced. In patients with frequent episodes of peritonitis, a perforation area due to PD catheter which limited itself should be considered as the etiology.

## 1. Introduction

Peritoneal dialysis (PD) catheters are placed in the abdomen using fluoroscopy by interventional nephrologists and interventional radiologists. Peritoneal dialysis is one of the most effective treatment options for end-stage renal failure patients worldwide [1, 2]. The procedure is straightforward, yet has the potential for complications. It is important to know how to prevent these complications, recognize them early, and manage them when they occur. Although peritoneal dialysis catheter placement is an outpatient surgical procedure, there are many complications associated with this procedure. Although peritoneal dialysis catheter placement is practically a daily surgical procedure, there are complications associated with surgical placement. While the early complications are infection, hematoma, leakage, and peritonitis, mostly related to comorbidities such as uncontrolled diabetes mellitus and poor nutrition, late complications are frequently caused by peritonitis attack. Early complications are infection, hematoma, leakage, and peritonitis, mostly due to comorbidities such as uncontrolled diabetes mellitus and malnutrition. Late complications are often caused by recurrent episodes of peritonitis. Additionally, there are few number of reports of intestinal and bladder perforation during the surgical procedure. Perforation cases have been reported months or even years after catheter placement [3, 4].

Delayed intestinal erosion with peritoneal dialysis catheter is rare, and less than thirty cases have been reported in the literature [5]. In this case report, we aimed to present a case with Tenckhoff catheter migration to the sigmoid colon after catheter replacement.

## 2. Case Presentation

A 53-year-old female patient was consulted to our clinic for revision of Tenckhoff catheter. When the medical records of the patient were checked, it was known that she had coronary artery disease and type 2 diabetes mellitus; she used 100 mg of acetylsalicylic acid and 2 g of pioglitazone per day, and she did not have a significant metabolic, psychosocial, or family history. The patient did not smoke. Tenckhoff catheter was applied to the patient with Seldinger technique two years ago. In these 2 years, the patient had repeated hospital admissions due to peritonitis attacks 4-6 times, and the first attack was 3 months after the catheter was inserted. These episodes of peritonitis were treated with antibiotics. During the initial physical examinations, the patient's general condition was good, vitally stable, conscious, and cooperative. Abdominal examination was normal.

Under general anesthesia, the patient's peritoneal dialysis catheter was replaced by laparotomy. On the first postoperative day, the patient developed abdominal pain, tachycardia, and general condition deterioration. On physical examination, there was tenderness and rebound in the bilateral lower quadrants. The patient was followed up in the intensive care unit. Intravenous fluid support and antibiotherapy (piperacillin-tazobactam) were started. Upon the development of acute abdomen in the follow-up of the patient, emergency exploration was planned for the patient.

**Figure 1:**
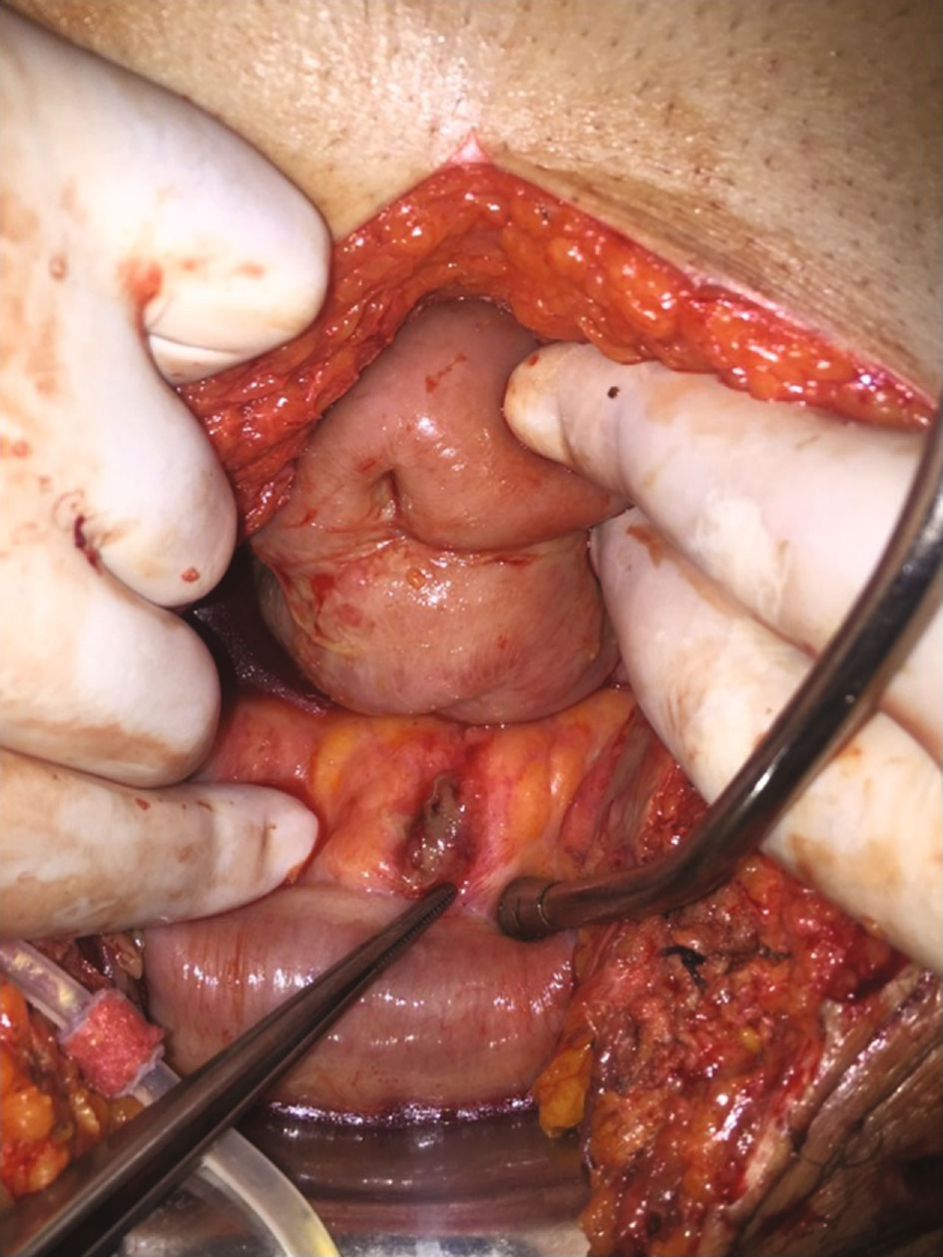
Entry from the sigmoid colon mesocolon.

**Figure 2:**
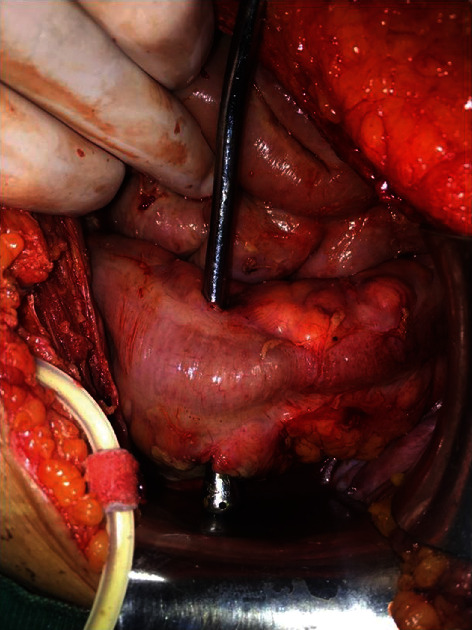
Perforated localization of the sigmoid colon.

On exploration, purulent drainage was observed in the pericolic region and it was seen that the last inserted peritoneal dialysis catheter passed transcolonic from the mesenteric surface of the sigmoid colon. When the existing catheter was removed, it was seen that this area was perforated (Figures 1 and 2). The newly inserted catheter did not cause perforation. Perforated area in the sigmoid colon was repaired with 3-0 silk (Silk; Dogsan Medical Supplies Industry, Trabzon, Turkey) with double layer sutures, and the abdomen was irrigated with abundant warm normal saline. The operation duration was 1 hour 20 minutes, and the estimated blood loss was 50 ml. A drain was placed in the Douglas pouch. The last introduced Tenckhoff catheter was also withdrawn.

The patient was followed up postoperatively in the intensive care unit, in which she was treated with antibiotic therapy (piperacillin-tazobactam), intravenous fluid, analgesics (paracetamol), and antiemetics. The patient's oral intake was started after the bowel movement was achieved. The patient had complete oral food intake on the 5th day. The drain of the patient was removed on the third day. Afterwards, the patient was transferred to the nephrology clinic for the planning of hemodialysis treatment.

The patient had no significant postoperative or wound complications and no significant long-term morbidity/mortality. Reexploration was not necessary. The patient was discharged from the nephrology clinic after the 21-day hemodialysis program was adjusted.

The patient was followed up for 6 months after discharge, and she did not have any complaints. Written informed consent was obtained from the patient.

## 3. Discussion

Success in peritoneal dialysis has been shown to be possible by providing a safe and permanent route to the peritoneal cavity. Although there have been improvements in catheter life duration in recent years, complications that still require catheter removal and affect the life of peritoneal dialysis are important problems [6].

Complications during peritoneal dialysis may interrupt treatment and may require hemodialysis. Previous studies with percutaneous technique have reported very low perforation rates (0-1.3%) [7, 8]. In our case, we think that the peritoneal dialysis catheter, which was inserted percutaneously 2 years ago, was placed transcolonically. From the patient's recurrent episodes of peritonitis, we speculate that colon wall erosion and microperforations caused by this catheter are responsible. We think that when we renew the existing catheter, the colon wall supporting the catheter is now free and the perforation area is more obvious and is responsible for the development of acute abdomen clinic.

Bowel perforation typically occurs at the time of PD catheter insertion. Delayed bowel perforation usually involves a dormant PD catheter. A long duration of a PD catheter in the abdominal cavity without peritoneal fluid, which bathes the bowel loops acting as a barrier of adhesion of the catheter to the bowel wall, increases the risk of pressure-induced necrosis by the immobile catheter. In addition, several potential risk factors for perforation were noted. Enlarged kidneys due to polycystic kidney disease occupying most of the abdominal cavity space might result in elevated intra-abdominal pressure. This might induce indolent erosion of PD catheters into the bowels. When the lower cuff of the double-cuffed catheters migrates into the peritoneal cavity, adhesion of the cuff to the intestinal wall may be another mechanism of bowel perforation. It has been suggested that the pathogenesis of delayed perforation in the intestine involves close contact between the peritoneal catheter and the intestinal wall. Continuous pressure causes local ischemia, causing erosion, laceration, and perforation [9].

The clinical signs of delayed bowel perforation of PD catheters are heterogenous and include symptoms such as peritonitis, watery diarrhea, catheter protrusion from the anus, and feculent discharge from the catheter [10]. In early perforations, contamination of the fluid given and taken during dialysis with bowel content suggests bowel injury. In the literature, many patients with delayed perforation have had a history of previous episodes of peritonitis [10, 11]. Our case had previous peritonitis attacks. In our case, perforation showed itself with acute abdomen and peritonitis after catheter replacement.

Treatment options range from a conservative approach that includes the catheter removal only, to a laparotomy with colonic resection and anastomosis [3, 5, 12]. There are reports in the literature that even the removal of the catheter alone is sufficient treatment without bowel repair, in patients without signs of sepsis or peritonitis [5, 12]. In our case, we did not prefer a minimally invasive approach because of the acute abdomen presentation. The fibrotic borders of the insertion site were resected, and primary closure was performed.

Delayed perforation due to peritoneal dialysis catheter may remain silent until it is detected after catheter replacement. Intestinal erosions due to peritoneal dialysis catheter should be considered in patients with frequent episodes of peritonitis. Early diagnosis and proper management of bowel perforations can be life-saving.

## Data Availability

The data that support the findings of this study are available from the corresponding author, [Ugur Topal), upon reasonable request.

## References

[1] Güllü B. E., Kahvecioğlu S. (2011). Comparıng catheter placement technıques by complıcatıons and dıalyıs effıcacy ın perıtoneal dıalysıs. *Journal of Istanbul Faculty of Medicine*.

[2] Li P. K., Chow K. M., Van de Luijtgaarden M. W. (2017). Changes in the worldwide epidemiology of peritoneal dialysis. *Nature Reviews Nephrology*.

[3] Ruiz J. Q. (2017). Erosion of a Tenckhoff catheter to the sigmoid colon: an uncommon delayed complication. *CEN Case Reports*.

[4] Kerwin L., Calhoun S., Calhoun S. (2015). Delayed intraperitoneal catheter erosion into the small bowel. *Case reports in radiology*.

[5] Vincent P., Gopinathan J., Narayanan R. (2017). Bowel migration of dormant chronic ambulatory peritoneal dialysis catheter: a vexed problem not avoided by flushing. *Indian Journal of Nephrology*.

[6] Koç Y., Yetkin G., Baştürk T., Uludağ M., Ahbap E. (2010). Comparison of the early complications of two different catheter implantation techniques. *Turkish Nephrology Dialysis And Transplantation Journal*.

[7] Moreiras Plaza M., Cuina L., Goyanes G. R., Sobrado J. A., Gonzalez L. (1999). Mechanical complications in chronic peritoneal dialysis. *Clinical Nephrology*.

[8] Zappacosta A. R., Perras S. T., Closkey G. M. (1991). Seldinger technique for Tenckhoff catheter placement. *ASAIO Transactions*.

[9] Kagan A., Bar-Khayim Y. (1996). Delayed decubitus perforation of the bowel is a sword of damocles in patients on peritoneal dialysis. *Nephron*.

[10] Handa T., Suzuki H., Matsubara H., Terajima H., Tsukamoto T. (2019). Unusual communication of an embedded peritoneal dialysis catheter with the colon before use: a case report with literature review. *Renal Replacement Therapy*.

[11] Chu P. Y., Siu K. L. (2016). A rare but serious complication of continuous ambulatory peritoneal dialysis: delayed perforation of the colon by the Tenckhoff catheter. *Hong Kong Medical Journal*.

[12] Wang R., Chen Z., Wang J., Zhang X., Shou Z., Chen J. (2014). Delayed bowel perforation in a peritoneal dialysis patient: a case report and literature review. *Peritoneal Dialysis International*.

